# Regulation, Communication, and Functional Roles of Adipose Tissue-Resident CD4^+^ T Cells in the Control of Metabolic Homeostasis

**DOI:** 10.3389/fimmu.2018.01961

**Published:** 2018-08-30

**Authors:** Haiyan Zhou, Feng Liu

**Affiliations:** ^1^Department of Metabolism and Endocrinology, Metabolic Syndrome Research Center of Central South University, The Second Xiangya Hospital, Central South University, Changsha, China; ^2^Department of Pharmacology, University of Texas Health Science Center at San Antonio, San Antonio, TX, United States

**Keywords:** obesity, CD4^+^T cells, antigen-presenting cells, inflammation, insulin resistance, energy homeostasis

## Abstract

Evidence accumulated over the past few years has documented a critical role for adipose tissue (AT)-resident immune cells in the regulation of local and systemic metabolic homeostasis. In the lean state, visceral adipose tissue (VAT) is predominated by anti-inflammatory T-helper 2 (Th2) and regulatory T (Treg) cell subsets. As obesity progresses, the population of Th2 and Treg cells decreases while that of the T-helper 1 (Th1) and T-helper 17 (Th17) cells increases, leading to augmented inflammation and insulin resistance. Notably, recent studies also suggest a potential role of CD4^+^ T cells in the control of thermogenesis and energy homeostasis. In this review, we have summarized recent advances in understanding the characteristics and functional roles of AT CD4^+^ T cell subsets during obesity and energy expenditure. We have also discussed new findings on the crosstalk between CD4^+^ T cells and local antigen-presenting cells (APCs) including adipocytes, macrophages, and dendritic cells (DCs) to regulate AT function and metabolic homeostasis. Finally, we have highlighted the therapeutic potential of targeting CD4^+^ T cells as an effective strategy for the treatment of obesity and its associated metabolic diseases.

## Introduction

Obesity, which is associated with various metabolic and cardiovascular diseases such as insulin resistance, type 2 diabetes, hypertension, and stroke, is among the most severe health threats to modern society (1, 2). With excessive nutrient intake and/or reduced energy expenditure, obesity triggers a state of chronic low-grade inflammation in the adipose tissue (AT). Specifically, visceral adipose tissue (VAT) is more prone to obesity-induced inflammation (3). Under the condition of overnutrition, the composition, number, and function of AT-resident immune cells are significantly altered, especially in white adipose tissue (WAT). Recent studies have demonstrated a dynamic crosstalk between adipocytes and immune cells, including both innate and adaptive immune cells, within AT. In response to nutritional or other environmental stimuli, altered AT-resident immune cells may initiate a low-grade inflammatory process, leading to insulin resistance and impaired metabolic homeostasis. Understanding the mechanisms underlying immune cell-initiated inflammatory responses in AT of obese individuals is thus of great clinical importance.

Naïve CD4^+^ T cells are developed in the thymus and then reside in secondary lymphoid organs such as spleen and lymphocytes and non-lymphoid organs such as AT (4). After activation by antigen-presenting cells (APCs), naïve CD4^+^ T cells may differentiate into one of the several lineages of T helper (Th) cells, including T-helper 1 (Th1), T-helper 2 (Th2), T-helper 17 (Th17), and regulatory T (Treg) cells, as defined by their specific patterns of cytokine production and function. Compared with secondary lymphoid tissues, AT contains few naïve CD4^+^ T cells but a large proportion of effect memory T cells that can regulate adaptive immunity based on interactions with APCs (5, 6). Indeed, AT-resident CD4^+^ T cells are one of the immune cells that rapidly respond to HFD challenge (7). However, the roles of AT-resident CD4^+^ T cells in metabolic homeostasis are not well established. In the lean state, WAT is dominated by anti-inflammatory Th2 and Treg cells, which help to maintain an anti-inflammatory milieu and metabolic homeostasis. The total number of CD4^+^ T cells in WAT is significantly increased after HFD feeding. However, as obesity progresses, the populations of Th2 and Treg cells are decreased (8), concurrently with increased Th1 and Th17 cells (9, 10). While accumulative studies have demonstrated a critical role of CD4^+^ T cells in obesity-induced inflammation, their roles in adaptive thermogenesis in subcutaneous WAT (SAT) and brown adipose tissue (BAT) remain in its infancy.

It is well documented that CD4^+^ T cell activation is initiated by antigen presentation. However, how AT-resident CD4^+^ T cells are activated during obesity remains controversial. There is some evidence suggesting that adipocytes, macrophages, and dendritic cells all could act as APCs to promote CD4^+^ T cell activation in AT (11–13).

In this review, we discuss recent findings on how AT-resident CD4^+^ T cells are involved in the regulation of local and systemic metabolic homeostasis. We also attempt to highlight the therapeutic potentials of targeting CD4^+^ T cells to treat obesity and its associated metabolic syndrome.

## Orchestration of CD4^+^ T cell subsets in immune responses

As an important component of adaptive immunity, CD4^+^ T cells play critical roles in defending against a large variety of pathogens. Besides, they are also involved in the pathogenesis of autoimmune diseases, asthma, and allergic responses. Naïve CD4^+^ T cells are activated by two signals including Class II major histocompatibility complex (MHCII)-mediated antigen presentation and co-stimulatory molecule- mediated co-stimulation, both provided by APCs. After activation, CD4^+^ T cells are differentiated into distinct subsets, depending on the cytokine signals in the microenvironment.

The four major lineages of CD4^+^ T cells, including the classical Th1 and Th2 cells, as well as Th17 and Treg cells, each have a characteristic cytokine profile (14). IL-12 and IFN-γ induce high expression levels of the Th1 master regulator T-box expressed in T cells (T-bet) and signal transducer and activator of transcription 4 (STAT4), promoting the naïve CD4^+^ T cells to differentiate into Th1 cells. With robust production of IFN-γ, Th1 cells mediate immune responses against intracellular pathogens (15). On the other hand, IL-4 induces high-level expression of STAT6 and GATA binding protein 3 (GATA3) in naïve CD4^+^ T cells, facilitating the differentiation of naïve CD4^+^ T cells to Th2 cells. With high expression levels of IL-4, IL-5, and IL-13, Th2 cells mediate host defense against extracellular parasites including helminths (15). Inappropriate Th2 responses are the major cause of allergic diseases such as asthma (16). IL-17-producing Th17 cells, induced by TGF-β, IL-6, IL-23, and IL-1β, contribute to the host defense against fungi and extracellular bacteria, with the high expression of their master regulator retinoic acid receptor-related orphan receptor-γt (RORγt) (15). The pathogenicity of Th1 and Th17 cells has been recognized in various autoimmune diseases, such as multiple sclerosis and rheumatoid arthritis (17, 18). Treg cells represent a subset of CD4^+^ T cells characterized by high suppressive capacity. As a key transcription factor of Treg cells, Foxp3 is indispensable for Treg cell development. Treg populations have also been identified and characterized in other non-lymphoid tissues such as skin, intestine, lung, liver, fat, muscle and placenta, clearly indicating an important role of Treg cells in the maintenance of tissue homeostasis (2, 19).

Cross-regulation among CD4^+^ T cell subsets by specific cytokine networks and transcription factors is critical for determining CD4^+^ T cell fates (14). Indeed, T-bet^−/−^mice exhibit a severe disease after virus infection and display asthma-like phenotype independent of allergen exposure (20). In addition, T cell-specific deletion of Gata3 results in impaired Th2 differentiation, permitting Th1 differentiation in the absence of IFN-γ and IL-12 (21). Similar to the crosstalk between Th1 and Th2 cells, cross-regulation has also been reported between Th17 and Th1 or Th2 cells. Both Th1-specific cytokine IFN-γ and Th2-specific cytokine IL-4 inhibit Th17 differentiation and induction of IL-17 (22). The immune homeostasis of Th1, Th2, and Th17 cells has also been found to be regulated by Treg cells via production of TGF-β and IL-10 (14).

## Roles of VAT-resident CD4^+^ T cells in obesity

### Treg cells

Treg cells are thought to be one of the most crucial defenses against inappropriate immune responses including autoimmunity, allergy, inflammation, and infection (23). Treg cells are highly enriched in the VAT of lean mice, but their numbers in this fat depot are markedly and specifically reduced in animal models of obesity and insulin resistance (2, 8). Treg cells contribute to the maintenance of insulin sensitivity in WAT by limiting inflammation and producing insulin-sensitizing factors such as IL-10 (24). IL-10 suppresses the expression of monocyte chemotactic protein-1 (MCP-1) in adipocytes to limit M1 macrophage infiltration of WAT. Besides, IL-10 could inhibit the ability of TNF-α to downregulate glucose transporter 4 (GLUT-4) expression and impair insulin action in adipocytes (25). A loss-of-function experiment by diphtheria toxin receptor (DTR)-mediated depletion of Treg cells and a gain of function experiment by injection of recombinant IL-2 and particular an IL-2-specific monoclonal antibody (mAb) have revealed that manipulating Treg cells can affect the inflammatory state of AT (2, 26).

Treg cell homeostasis in VAT is regulated by iNKT cells via the production of IL-2 (27). Besides, IL-33, which is mainly secreted by a number of different stromal cell types including Cadherin11^+^ (Cdh11^+^) mesenchymal cells, podoplanin^+^ (Pdpn^+^) fibroblasts, and CD31^+^ endothelial cells, is required for Treg cell accumulation in VAT through binding to its receptor Interleukin 1 receptor-like 1, also known as IL1RL1 or ST2 (28, 29). In addition, IL-33 and IFN-γ counter-regulate group 2 innate lymphoid cells (ILC2) activation to control Treg cell numbers (30). The control of Treg cells by ILC2 is independent of the cytokines of ILC2 but mediated by a direct interaction of co-stimulatory molecules inducible co-stimulator (ICOS) and ICOS ligand (ICOSL) (30).

What is the origin of AT-resident Treg cells? It is reported that the AT-resident Treg and conventional T (Tconv) cell populations have different repertoires, suggesting that the accumulation of Foxp3^+^ Treg cells in VAT is not due to the local conversion of Tconv cells (2). The VAT-resident Treg cells are also found not to be originated from circulating Treg cells. On the other hand, there is strong evidence suggesting that VAT-resident Treg compartment comes from thymus and their accumulation depends on interactions with local APCs (28).

VAT-resident Treg cells have a distinct transcriptome and antigen-receptor repertoire from those of their counterparts in the spleen and lymph nodes (31). Notably, peroxisome proliferator-activated receptor (PPAR-γ), the master regulator of adipocyte differentiation, is identified as a crucial molecular orchestrator of VAT Treg cell accumulation, phenotype, and function (31). Specifically, knockout of PPAR-γ in Treg cells significantly lowered the fractions and numbers of Treg cells in VAT but not in lymphoid organs. The thiazolidinedione (TZD) drug pioglitazone (Pio), a well-known insulin-sensitizing agent, is a synthetic agonist for PPAR-γ. Pio treatment specifically promotes VAT-resident Treg cell numbers and phenotype in HFD-fed wild-type (WT) mice but not in PPAR-γ mutant mice (31).

### Th2 cells

Similar to VAT-resident Treg cells, the IL-4- and IL-13-expressing Th2 cells accumulate in VAT of older animals. Compared with VAT-resident Treg cells, the numbers of VAT-resident Th2 cells are relatively rare and their function in obesity progression is much less studied. VAT Th2 cells also express ST2 and treatment of ob/ob mice with IL-33 leads to the production of strong Th2 cytokines in WAT, resulting in improved insulin sensitivity (32, 33).

Adoptive transfer of CD4^+^ T cells into HFD-fed Rag1-null mice has normalized obesity-associated insulin resistance (34). Interestingly, the beneficial effects of CD4^+^ T cells in the adoptive transfer model are found to be contributed by Th2 cells but not Treg cells, since mice transferred with Foxp3^−−/−^ or IL-10^−−/−^ CD4^+^ T cells show no obvious changes in the phenotypes compared with mice transferred with WT CD4^+^ T cells. By contrast, reconstitution with STAT6^−/−^ CD4^+^ T cells leads to the loss of the insulin-sensitizing effects of the WT CD4^+^ T cells, suggesting that Th2 cells are important controllers of obesity and insulin resistance (34).

### Th1 cells

Under over-nutrition conditions, VAT expansion creates an environmental milieu that potentiates the influx of proinflammatory cells and the production of type 1 cytokines such as IL-6, TNF-α, IL-1β, and IFN-γ (35, 36). The immune homeostasis in VAT is consequently disrupted with a decrease of Treg and Th2 cell populations, concurrently with a significant increase of proinflammatory T cells, especially IFN-γ-producing Th1 cells, CD8^+^ T cells, and Th17 cells (9, 10, 12, 37, 38). Consistent with these findings, T-bet deficient mice display enhanced insulin sensitivity though increased VAT mass (39), suggesting that deficiency of Th1 cells may lead to metabolic restoration. Increasing Th1 cells accelerate insulin resistance by producing TNF-α and IFN-γ in WAT (11). IFN-γ is a robust proinflammatory cytokine that activates M1 macrophages (40), promotes Th1 cell polarization (41), and induces inflammatory mediators such as MHCII (9, 42). The mRNA level of IFN-γ rapidly increases even after just 1-week HFD feeding (42). High level of IFN-γ in human VAT is also associated with increased waist circumference (7). IFN-γ-deficient mice show a significant decrease in inflammatory gene expression and accumulation of leukocytes, as well as improved glucose tolerance (9). Taken together, these findings reveal that decreased IFN-γ levels and/or Th1 cell expansion in AT are beneficial for suppressing inflammation and improving insulin sensitivity.

### Th17 cells

Th17 cells are usually regarded as proinflammatory cells. However, the role of AT-resident Th17 cells in obesity remains largely elusive. One study shows that, although the total number of Th17 cells in VAT remains unchanged, the percentage of Th17 cells is decreased during the development of obesity (34). To the contrary, other studies show that the numbers or percentages of Th17 cells are increased in AT of obese humans compared to their lean controls (12, 43). Th17 cells have been suggest to block insulin receptor signaling and contribute to metabolic dysfunction via promoting the secretion of IL-17 and IL-22 (43–45). HFD feeding is also able to stimulate splenic Th17 cell development, thus accelerating the onset of autoimmune diseases such as experimental autoimmune encephalomyelitis (EAE) and collagen-induced arthritis (CIA) (10, 46). Mechanistically, obesity boosts Th17 cell polarization by upregulating Acetyl-CoA carboxylase 1 (ACC1) expression, which promotes the binding of RORγt to the IL-17 gene locus (10). RORγt^−/−^ mice fed a normal-chow diet display glucose intolerance hyperinsulinemia, and slightly insulin resistance. However, RORγt^−/−^ mice fed a HFD rapidly lost weight and die within 1 month, probably due to a deleterious effect of the lipotoxicity. Of note, after HFD feeding, heterozygous RORγt^+/−−^ mice have impaired glucose tolerance and increased insulin resistance compared with WT control mice (47).

IL-17, a major effector cytokine produced by Th17 cells, functions as a negative regulator of adipogenesis (48, 49). IL-17 deficiency enhances diet-induced obesity in mice and accelerates fat mass accumulation even in mice fed a low-fat diet (49). It has been shown that γδ T cells, but not Th17 cells, are the predominant cells that produce IL-17 in WAT under both ND and HFD conditions (49, 50). Thus, the role of IL-17 is not equal to the role of Th17 cells under either ND or HFD conditions. Mice lacking γδ T cells or IL-17A had impaired ability to regulate core body temperature at thermoneutrality and after cold challenge due to the decreased ST2^+^ Treg cells and IL-33 abundance in AT (50). Nevertheless, the function of Th17 cells in obesity-related WAT inflammation is complex and requires further investigation.

## CD4^+^ T cells crosstalk with APCs

CD4^+^ T cells in VAT are increasingly recognized as a key regulator of AT inflammation and systemic insulin action. APCs are indispensable for the activation and differentiation of CD4^+^ T cells (11, 51). Importantly, VAT resident CD4^+^ T cells, regardless of their specific lineages, demonstrate distinct and selective T cell receptor (TCR) repertoires compared with their circulating counterparts, suggesting an AT-specific antigen expansion (2, 31, 34). This finding further strengthens the view that APCs also exist in AT and are required for AT-resident CD4^+^ T cell polarization. Although the exact nature of the antigens is still largely unknown, recent studies have revealed different types of APCs including macrophages, adipocytes and dendritic cells in the course of obesity-related AT inflammation (11, 42, 52).

### Adipose tissue macrophages (ATMs)

Macrophages have been implicated as one of the important types of APCs found in AT (11). Based on their characteristics and functions, adipose tissue macrophages (ATMs) can be categorized into “classically activated” M1 macrophages and “alternatively activated” M2 macrophages (36). Obesity is accompanied by a switch in macrophage activation from the protective M2 macrophages to the proinflammatory M1 macrophages (53). ATMs are thought to be the predominant MHCII-expressing cells in VAT under both ND and HFD feeding conditions (51). MHCII-deficient mice are protected from HFD-induced insulin resistance with the reduction of ATMs and CD4^+^ T cells accumulation in VAT (11, 42). It is believed that AT-resident M2 macrophages are the predominant APCs in lean mice and humans (11, 36). MHCII in M2 macrophages is required to translate obesogenic cues into CD4^+^ T cell immune responses at the initial stage of obesity (11). During the development of obesity, M2 macrophages may progressively obtain a proinflammatory phenotype and induces Th1 cell polarization, accelerating the development of insulin resistance (54). Immunofluorescence and intravital imaging analysis show that ATMs physically interact with CD4^+^ T cells in an antigen-dependent manner (11). Macrophage-specific deletion of MHCII has no effect on AT-resident T cells in the lean state, but significantly prevents the generation of effector memory AT-resident CD4^+^ T cells and insulin resistance in AT (11). Diet-induced obesity also promotes the expression of T-cell co-stimulatory molecules, such as CD80 and CD40, on the surface of ATMs in VAT (55). CD40 deficiency affects ATM infiltration into VAT and decreases T cell accumulation during diet-induced obesity.

In contrast to the classical view of ATMs being grouped into M1 and M2 macrophages, a recent study shows that CD9 and Ly6c define unique populations of ATMs in obese AT, with CD9 ATMs predominating in crown-like structures (CLS) and Ly6c ATMs uniformly distributed in AT (56). CD9 ATMs contain high levels of intracellular lipid and express proinflammatory transcriptomes while Ly6c ATMs express factors that support angiogenesis and tissue organization (56). In addition, adoptive transfer of CD9 ATMs, but not Ly6c ATMs, from obese donor mice to lean recipients confers an inflammatory response to the AT of lean mice (56). Nevertheless, whether these two subsets of ATMs function distinctively in antigen presentation is not explored. Since MHCII expression ATMs are concentrated in CLS in obese AT (51), There is a possibility that CD9 ATMs, rather than Ly6c ATMs, may be the main ATMs that activate CD4^+^ T cells in AT. Further studies are needed to address this possibility.

### Adipocytes

While the role of ATMs in AT inflammation is well documented, several studies report that macrophages do not infiltrate into AT until 10 weeks after HFD feeding (7, 42). Indeed, CD8^+^ effector T cells are believed to contribute to the later macrophage recruitment (37). On the other hand, an early infiltration of lymphocytes is observed soon after HFD feeding (7, 37, 42). The early presence of T cells in VAT at the time of manifest insulin resistance raises a possibility that there may be other APCs that initiate T cell activation in AT. Consistent with this view, adipocytes are recently implicated as APCs that influence T cell activation in obesity (42). Expression of MHCII in adipocytes begins to increase within 2 weeks of HFD challenge, paralleled with early changes of AT-resident CD4^+^ T cells which show enhanced expression of the proinflammatory Th1 marker genes (42). HFD-fed MHCII^−/−^ mice show less adipose inflammation and insulin resistance (42). Mechanically, it is suggested that, as obesity advances, leptin secreted by adipocytes stimulates IFN-γ production from CD4^+^ T cells, which further promotes adipocyte MHCII expression and thus Th1 differentiation, leading to a vicious cycle of AT inflammation (42). The specific contribution of this vicious cycle to metabolic dysfunction is further verified by adipocyte-specific disruption of MHCII. AT-specific knockout of MHCII suppresses AT IFN-γ production and increases Treg accumulation, leading to reduced AT inflammation and insulin resistance in obese mice (13). Inhibition of MHCII expression in adipocytes by adrenomedullin 2 treatment restores the HFD-induced early insulin resistance due to decreased CD4^+^ T cell activation (57). It is suggested that IL-10 produced by adipocytes may dampen the APC function of ATMs, thus showing the superiority of adipocytes over ATMs as APCs at the early stage of obesity (42). Indeed, recruitment of M1 macrophages into WAT is induced by inflammatory mediators such as MCP1, C-X-C motif chemokine 12 (CXCL12) produced by dead and neighboring adipocytes (58, 59). While APCs may shape CD4^+^ T cells, CD4^+^ T cells can also influence the recruitment and activation of APCs. CD40L is induced in AT CD4^+^ T cells after HFD feeding, which can further stimulate activation of ATMs as well as adipokine production of adipocytes through ligation with CD40 (55, 60).

Adipocytes secret various adipokines such as leptin, adiponectin, and resistin, which are implicated in the regulation of CD4^+^ T cell immune responses. Leptin receptor is expressed in human AT T cells and its expression increases with obesity (61). Impairment of leptin receptor signaling improves Treg cell immune responses (62, 63). However, how does leptin signaling regulate Treg responses remains elusive. Although both IL-33 and ILC2 are found to promote AT Treg accumulation (30), studies show that IL-33 expression positively correlates with leptin expression in human AT (64). Obesity-associated elevation of leptin also contributes to the increased susceptibility of asthma via modulation of Th2 and ILC2 response (65). These findings suggest that leptin may regulate Treg immune response independent of IL-33 and ILC2. On the other hand, leptin receptor signals are required for Th17 differentiation via activation of signal transducer and activator of transcription 3 (STAT3) and through cooperate with IL-6 (45, 66, 67). Leptin can also stimulate Th1 cell differentiation through promoting IFN-γ secretion (42, 68). Adiponectin is another adipokine that has been shown to directly enhance Th1 differentiation by activating the p38-STAT4-T-bet axis (69). Adiponectin activates DCs leading to enhanced Th1 and Th17 responses (70). Lastly, resistin has been found to stimulate CD4^+^ T cell chemotaxis in a concentration-dependent manner (71).

During obesity, bioactive lipids released by adipocytes also involve in the regulation of CD4^+^ T cells. Ceramide synthesis is elevated under obesity conditions, correlating positively with the degree of insulin resistance (72). Ceramides are localized predominantly within the cell membrane and are suggested to enhance Th1 cell differentiation together with IL-12 (73). Many ceramide derivatives have been found to inhibit IL-4 production in T cells (74). n-3 polyunsaturated fatty acids (PUFA), such as eicosapentaenoic acid (EPA) and docosahexaenoic acid (DHA), can alter the biochemical and biophysical properties of CD4^+^ T cell plasma membranes, thus modulating cytoskeletal dependent CD4^+^ T cell activation and differentiation (75). It is also suggested that n-3 PUFA suppress Th1/Th17 immune responses in diverse tissues in obese mice following the induction of colitis (76).

Whether adipocytes in WAT of lean mice also play a role as APCs is not clear. VAT Adipocytes of lean mice show low but detectable MHCII expression. When cocultured with CD4^+^ T cells *in vitro*, adipocytes of lean mice could stimulate CD4^+^ T cells IFN-γ and IL-2 production though to a much less extent than that of obese mice (42). The antigen-presentation capacity of adipocytes from lean AT-specific MHCII knockout mice is remarkably reduced compared with lean WT mice (13). It is possible that adipocytes of lean mice with low-level MHCII expression could also act as APCs. However, whether and how adipocytes of lean mice function as APCs to regulate CD4^+^ T cell activation *in vivo* remain to be further determined.

### Adipose tissue dendritic cells (ATDCs)

Dendritic cells (DCs) are professional APCs and play an important role in promoting CD4^+^ T cell activation and polarization (77). However, it has been difficult to clarify the contribution of ATDCs to AT inflammation since clear discrimination between ATDCs and ATMs in AT is limited. It is suggested that, in lean mice, the majority of CD11c^+^ cells are ATDCs but not ATMs (78). HFD feeding for 16 weeks led to a substantial increase in CD11c^+^ infiltrating M1 macrophages and the maintenance of a prominent population of CD11c^+^ ATDCs (78). Since ATMs and ATDCs are both CD11c^+^ cells in WAT of obese mice, macrophage-specific marker CD64 is thus adopted to distinguish the two populations, with CD11c^+^CD64^+^ identified as infiltrating M1 macrophages and CD11c^+^CD64^−^ identified as ATDCs (11). Both populations have similar capacities to stimulate CD4^+^ T cell proliferation (78).

Another study defines CD11b^−^CD11c^+^ cells as ATDCs, which express higher levels of MHCII than CD11b^+^CD11c^+^ ATMs (28). Confocal analysis reveals that both Treg and Tconv cells are in close contact with ATMs and ATDCs (28). The distance between T cells and APCs is dramatically increased in mice treated with an anti-MHCII mAb, suggesting that ATMs and ATDCs may contact with T cells through MHCII. (28). Ablation of CD11c^+^ cells by DTR normalizes insulin sensitivity in obese and insulin resistant mice (79). Since CD11c is commonly recognized as a marker of DCs, this finding suggests that the deletion of DCs, at least in part, may contribute to the increased insulin sensitivity (80).

The majority of ATDCs in the lean state are thought to be CD11c^high^F4/80^−^CD103^+^ cells. Since CD103^+^ DCs are able to induce the development of Treg cells (81), it is suggested that this CD11c^high^F4/80^−^CD103^+^ ATDCs play a role in the induction of AT Treg cells to restrain AT inflammation (12). Some atypical CD11c^high^F4/80^low^CX3CR1^+^ ATDCs are also detectable at a very low frequency (<1%) in the AT of lean mice. Both the frequencies and absolute numbers of these two ATDCs populations are increased after HFD feeding, accompanied by enhanced antigen-presenting abilities to induce Th17 differentiation (12). It's worth mentioning that the increased atypical CD11c^high^F4/80^low^CX3CR1^+^ ATDCs, regarded as inflammatory DCs in AT, are the major contributors to the induction of Th17 cells in AT of obese mice possibly via expressing high levels of IL-6, TGF-b, and IL-23 (12, 52). This observation is in accordance with previous studies that demonstrate the importance of obesity in the expansion of Th17 cells (10, 46).

Although much progress has been made on our understanding of the role of AT-resident CD4^+^ T cells in regulating metabolism, it is still unclear which cells are the major APCs at different stages of obesity and whether these APCs cooperate to activate CD4^+^ T cells. To define distinct populations within each APCs with unique transcriptomes and functions is of great importance, which will help to develop APCs-based therapies for the treatment of obesity and related inflammatory comorbidities.

## The roles of CD4^+^ T cells in energy homeostasis in SAT and BAT

Despite extensive studies on the functional roles of adipose-immune crosstalk in VAT, the role and regulation of CD4^+^ T cells in adaptive thermogenesis are much less clear. Several recent studies have uncovered a potential function of Treg cells in SAT and BAT in regulating energy homeostasis (4, 82). BAT-resident Treg cells share many similar characteristics with VAT-resident Treg cells, although BAT harbors more Treg cells than VAT (82). Systemic depletion of Treg cells impairs oxygen consumption under cold stimulation conditions (82). In fact, treatments known to enhance sympathetic tone and promote BAT thermogenesis such as cold exposure, short-term high-calorie input, and β-adrenergic stimulation, greatly increase Treg cells in WT but not in β-less mice in which all of the three β-adrenergic receptors are deleted (4). These results indicate an essential role for thermogenic response in BAT Treg cell accumulation. Indeed, UCP-1^−/−^ mice exhibit reduced Treg cells in BAT and SAT compared with WT control mice (4). Furthermore, loss-of-function and gain-of-function experiments all suggest that Treg cells are critical for BAT thermogenic capacity and lipolytic function (4). The T cell-specific Stat6/Pten axis is believed to link beta3-adrenergic stimulation to Treg cell induction in BAT and SAT, which is consistent with a previous report that inhibition of PI3K/AKT could promote Treg differentiation (4, 83). Interestingly, IL-17-producing γδ T cells are recently reported to regulate thermogenesis via BAT Treg cells, further supporting an important role of Treg cells in energy expenditure (50).

In contrast to the role of Treg cells, the role of Th2 cells in energy expenditure is largely unknown. However, given that both the transcription factors and cytokines are functionally similar between Th2 cells and ILC2s (32, 84), it is possible that the Th2 cells may also play a part in energy expenditure. Further investigations will be needed to address this question.

Rag1^−/−^ mice that lack both T and B lymphocytes display excess weight gain under HFD-feeding conditions, which is at least in part due to decreased energy expenditure resulted from decreased UCP-1 expression in BAT (85). In contrast, another study showed that even in the lean state, Rag1^−/−^ mice display more energy expenditure and upregulated of UCP1 expression in SAT than WT mice at room temperature (86). Decreased CD8^+^ T cells, but not CD4^+^ T cells, are believed to contribute to promote beige fat development, mainly due to the decreased IFN-γ secretion (86). However, given that Th1 cells are also major producers of IFN-γ and that HFD feeding increases both the percentage and the total number of Th1 cells in SAT, it is possible that Th1 cells may also be involved in the regulation of energy expenditure.

## Therapeutic implications of targeting CD4^+^ T cells

### Targeting chemokines and their receptors

Infiltration of proinflammatory CD4^+^ T cells into VAT is now recognized as one of the primary events in obesity-induced chronic inflammation. Chemokines and their receptors play crucial roles in the trafficking of leukocytes to lesions and areas of inflammation (87, 88). Antagonizing chemokines and/or their receptors by small molecules or antibodies have been shown to be another promising approach to suppress inflammation and potentially, improve metabolic dysfunction.

Indeed, CD4^+^ T cells, as well as CD8^+^ T cells and B cells, from ob/ob mice had a greater propensity to migrate specifically to inflamed tissues (89). The regulated on activation normal T cell expressed and secreted (RANTES), also known as CCL5, is a chemokine that plays an active role in recruiting leukocytes into inflammatory sites. RANTES and its chemokine receptor CCR5 have been implicated in T cell trafficking to VAT in the setting of murine and human obesity (88). The expression of RANTES and CCR5 in WAT, especially the SVF fraction, is increased in a gender-dependent fashion in obese mice (88). Interestingly, monoclonal antibodies against RANTES have been shown to significantly reduce T-cell chemotaxis (88, 90).

The CCR5/RANTES axis also plays an important role in the progression of hepatic inflammation and fibrosis. Maraviroc, a CCR5 antagonist that has already been approved by FDA for the treatment of human immunodeficiency Virus (HIV) (91), ameliorates hepatic steatosis in an experimental model of NAFLD (87). Another CCR5 ligand, CCL3, is also secreted at significantly high levels in the omentum of patients with an obesity and inflammation-driven cancer oesophagogastric adenocarcinoma (OAC). Antagonizing CCL3 receptor, including CCR5 and CCR1, significantly reduces T cell migration to the omentum of OAC patients (92). As obesity develops, human adipocytes release the chemokine CCL20 and promote T cell migration into VAT via its receptor CCR6 (61).

Pharmacological inhibition of chemokines may exert beneficial pleiotropic effects in several metabolically active organs since these organs are likely to be affected by similar cellular, molecular, or endocrine pathways (93). Elucidation of the mechanisms that recruit inflammatory CD4^+^ T cell to AT should improve our knowledge for developing novel therapeutics for inflammation-associated metabolic dysfunctions.

### Promoting treg cell accumulation in AT

**Figure 1:**
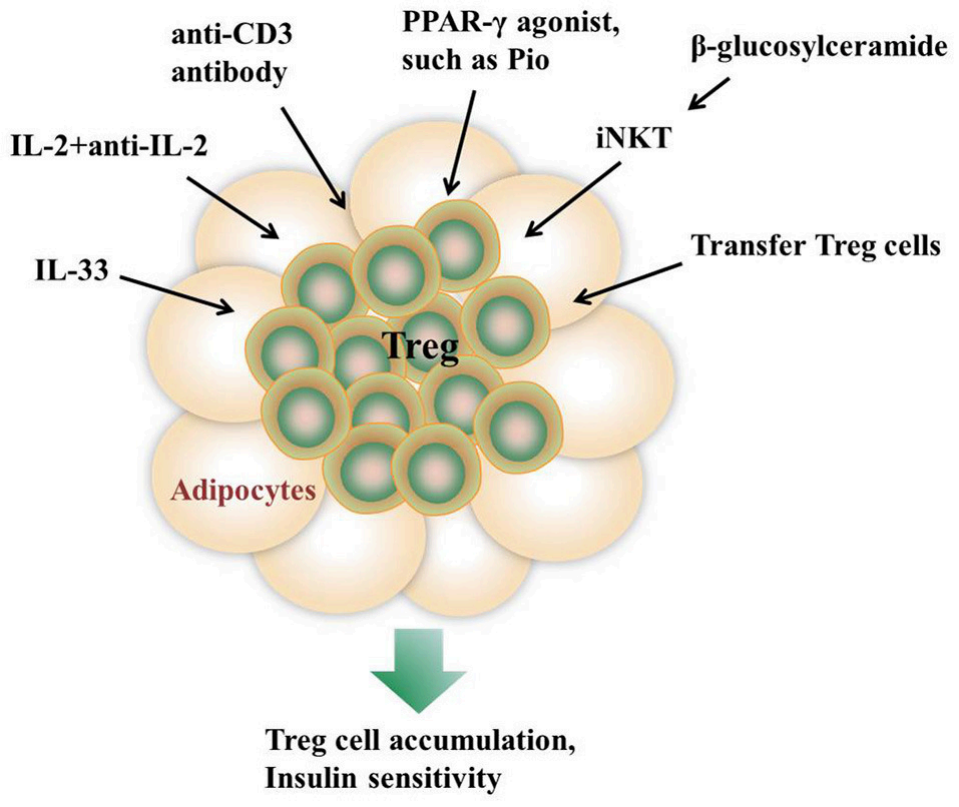
Approaches to promote AT Treg cell accumulation *in vivo*. Injection of IL-33, the anti-CD3 antibody, or a complex consisting of recombinant IL-2 and a particular anti-IL-2 monoclonal antibody all show a long-lasting effect on Treg cell expansion in both lymphoid tissues and VAT. β-glucosylceramide induces iNKT cells activation which promotes Treg cells expansion via IL-2 production. In addition, adoptive transfer of Treg cells into recipient mice is recognized as a straight way to increase Treg populations. PPAR-γ agonist Pio treatment also specifically promotes VAT Treg cell accumulation.

Induction of Treg cells is one of the major goals in immunotherapy of autoimmune diseases and transplantation. The emerging notion that Treg cells in AT are important for immune homeostasis and thermogenesis has evoked an exciting possibility to expand Treg cells as a therapeutic strategy for the treatment of obesity-induced metabolic dysfunctions (94) (Figure 1). In some studies, mitogenic anti-CD3 antibody is utilized to promote T cell self-tolerance through a global but transient T cell depletion, which leads to a selective increase of Treg cell pools at sites of tissue inflammation (95, 96). Injection of an anti-CD3 antibody to HFD-fed mice for 5 days greatly improves glucose tolerance and insulin sensitivity (34). In addition, the normalizing effects on insulin resistance and glucose tolerance last for over 4 months even under the condition of sustained HFD feeding, suggesting a long-lasting therapeutic effect (34). Oral administration of an anti-CD3 antibody plus β-glucosylceramide displays a decrease in pancreatic islet cell hyperplasia, fat accumulation in the liver, and inflammation in adipose tissue via induction of Treg cells (24). In addition, a complex consisting of recombinant IL-2 and a particular anti-IL-2 mAb is used to induce *in situ* expansion of Treg cells (2, 97). IL-33 injection shows a long-lasting effect on Treg cell expansion in both lymphoid tissues and VAT (28), while PPAR-γ agonist Pio treatment specifically promoted VAT Treg cell accumulation (31). In some studies, adoptive transfer of Treg cells into recipient mice is recognized as a straight way to increase Treg populations (4).

### Targeting gut microbiota

Profound gut microbiota alterations are found to be closely associated with obesity and metabolic syndrome in recent years (98). HFD feeding induces prominent alterations in the gut microbiota composition by increasing the Firmicutes to Bacteroidetes ratio, which positively correlates with body weights in humans (99–101). The development and maturation of CD4^+^ T cells are influenced by gut microbiota. Eating purified probiotic microbe alone, namely Lactobacillus reuteri, prevented weight gain irrespective of the baseline diet due to the promotion of IL-10-producing Treg cells (102). HFD-fed mice supplemented with a mixture of foodborne lactic acid bacteria show reduced VAT mass with increased Treg cells (103). There is some evidence showing that HFD-derived ileum microbiota is responsible for a decrease of Th17 cells in the lamina propria, while microbiota from synbiotic-treated obese mice increases the number of intestinal Th17 cells and improves glucose tolerance (47). In addition, delivery of Th17 cells to the intestines of obese mice leads to expansion of commensal microbes that maintain metabolic homeostasis (104). However, the precise mechanisms by which microbiome regulates CD4^+^ T cells and thus metabolic homeostasis remain largely unknown and require further investigations.

## Conclusions and perspectives

New evidence accumulated over the past several years strongly implicates an important role of AT-resident immune cells such as Th2 and Treg cells in the housekeeping functions of animals or humans via regulation of inflammation. During the progression of obesity, specific antigens in VAT are produced, captured, and presented to CD4^+^ T cells by APCs, leading to decreased Th2 and Treg cell populations and a shift to increased proinflammatory Th1 and Th17 cell populations. The timeline of appearance or changes of the major cell types or factors that contribute to the proinflammatory status of adipose tissue after HFD feeding is summarized in Table 1. Although the roles of CD4^+^ T cells in obesity have been largely investigated, more efforts will be needed to elucidate the function of CD4^+^ T cells in the regulation of energy expenditure in AT.

**Table 1:** Summary of the timeline of appearance or changes of the major cell types or factors that contribute to the proinflammatory status of adipose tissue after HFD feeding.

**Major cell types or factors**	**Timeline of appearance or changes**	**References**
Leptin	Within 1 week	(42)
Adipocyte MHCII	Within 2 weeks	(42)
T-bet	2 weeks	(42)
GATA3	3 weeks	(42)
Foxp3	12 weeks	(42)
IFN-γ	2 weeks	(42)
M1	10–12 weeks	(42), (7)
CD3	5 weeks	(7)
CD11c ^high^F4/80 ^low^	15 weeks	(12)

**Figure 2:**
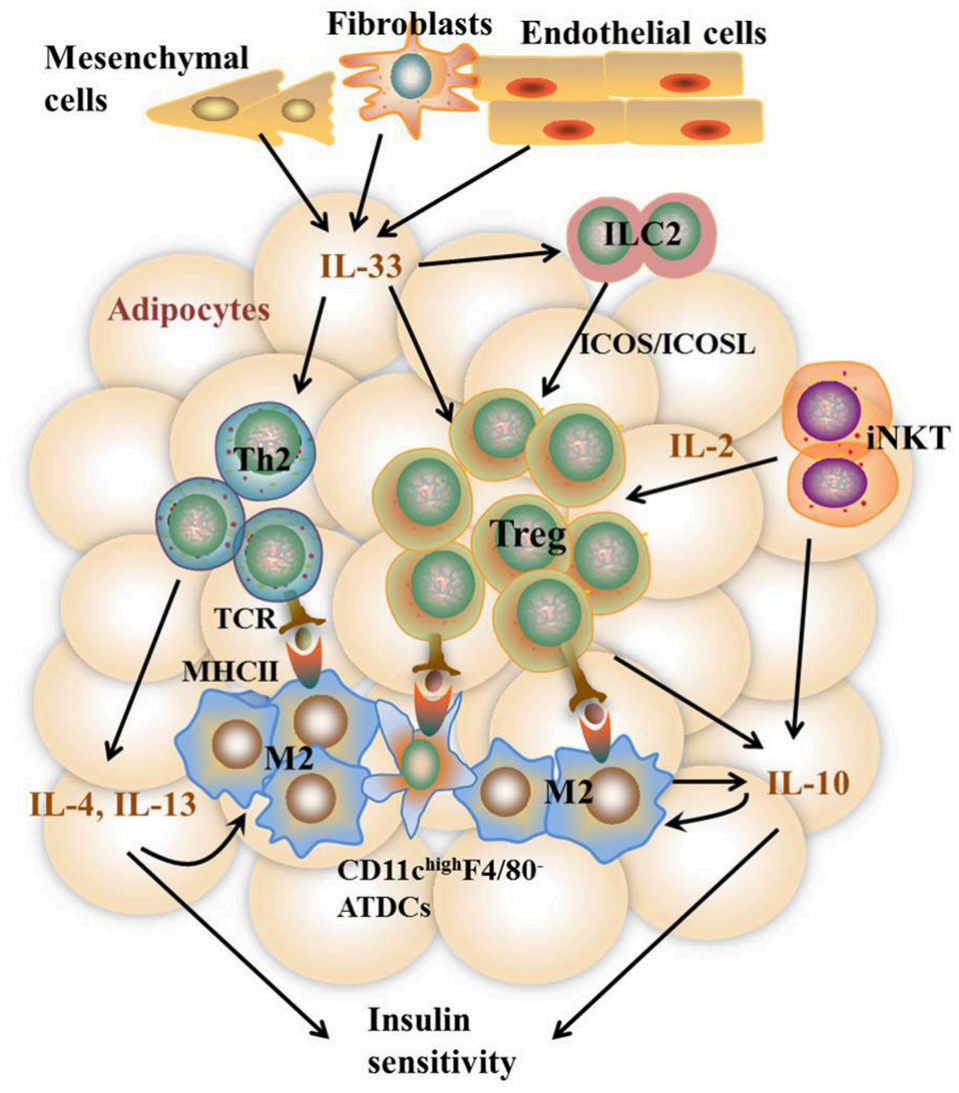
Th2 and Treg cell-mediated immune responses in VAT under the lean state. In the lean state, activation and proliferation of AT-resident Th2 and Treg cells are mediated by APCs including M2 macrophages, CD11c ^high^F4/80^−^ ATDCs, and maybe adipocytes with low MHCII expression. Activated Th2 cells produce type 2 cytokines including IL-4, IL-5, and IL-13, which together with iNKT and Treg cell-produced IL-10 further stimulate M2 activation. IL-10 also acts directly on adipocytes to promote insulin sensitivity. The homeostasis of Th2 and Treg cells is promoted by constitutively produced IL-33 from Cdh11^+^ mesenchymal cells, Pdpn^+^ fibroblasts, and CD31^+^ endothelial cells via high expression of ST2 on the surface of both cells. In addition, IL-33 and IFN-γ counter-regulate ILC2 activation to control Treg cell numbers. Besides, iNKT cells are necessary to sustain Treg cells via the production of IL-2. Together, these pathways contribute to metabolically healthy VAT.

**Figure 3:**
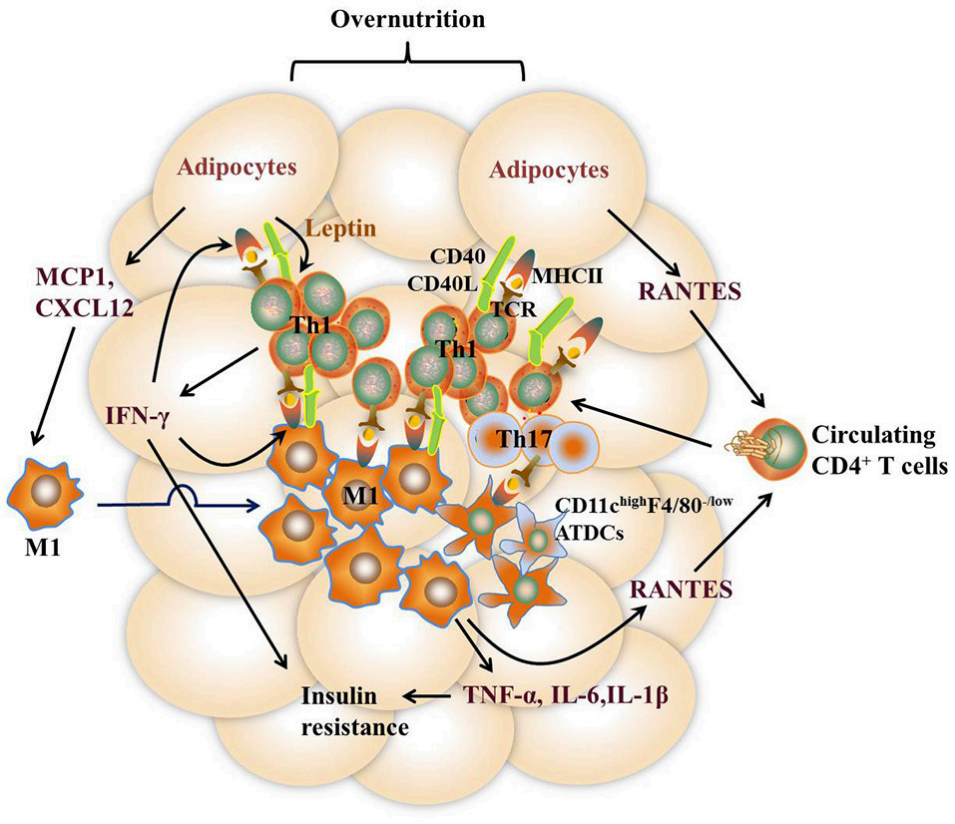
Th1 and Th17 cell-mediated immune responses in VAT under the obese state. Overnutrition causes adipocyte hypertrophy, leading to the release of chemokines such as RANTES, which recruit proinflammatory CD4^+^ T cell accumulation in VAT via its receptor CCR5. Leptin secreted by adipocytes stimulates IFN-γ production from CD4^+^ T cells, which further promotes adipocyte MHCII expression and antigen-presentation to induce Th1 cell differentiation, leading to a vicious cycle of AT inflammation. Dead and neighboring adipocytes recruit M1 macrophages to WAT by producing inflammatory mediators such as MCP1 and CXCL12. Likewise, the expression of MHCII in M1 macrophages is promoted by IFN-γ, thus facilitating M1 macrophage-mediated antigen-presentation to induce Th1 cell differentiation. In addition, diet-induced obesity also promotes the expression of inflammatory receptor CD40 expression on ATMs and adipocytes as well as CD40L on CD4^+^ T cells, which reinforce the crosstalk between CD4 ^+^ T cells and these APCs. CD11c ^high^F4/80 ^low^ ATDCs are also regarded as APCs to induce Th17 reactivation via production of TGF-β, IL-6, and IL-23. Type 1 cytokines such as TNF-α and IFN-γ, act directly on adipocytes to impair insulin action. Together with IL-6 and IL-1β, these cytokines elicit sustained chronic inflammation that eventually leads to insulin resistance.

It remains to be controversial as to which cell types are the major APCs in the AT. Both ATMs and adipocytes show enhanced MHCII gene expression under obesity conditions. Deficiency of MHCII in either macrophages or adipocytes shows improved metabolic phenotypes in mice (11, 13). However, several studies report that macrophages do not infiltrate into AT until 10 weeks after HFD feeding while MHCII family genes are upregulated at 2 weeks after HFD, indicating that adipocytes but not ATMs are the APCs that initiate T cells activation (7, 42). On the other side, several studies suggest that ATMs are the predominant MHCII-expressing cells in VAT under both ND and HFD feeding conditions, arguing that non-macrophage cells such as adipocytes play a minor role in MHCII expression in adipose tissue (11, 28, 36, 53). Thus, it is possible that different cell types may act as APCs at different stages of obesity. At early stages, adipocytes of lean mice with a low expression of MHCII, AT-resident M2 macrophages, and CD11c^high^F4/80^−^ ATDCs may act as APCs, leading to the homeostatic proliferation of Th2 and Treg cells. As obesity develops, adipocytes of obese mice with markedly increased MHCII expression, infiltrating M1 macrophages, as well as CD11c^high^F4/80^−^ and CD11c^high^F4/80^low^ ATDCs become dominant in the AT that act as APCs instead [Figures 2, 3). This notion, to a certain extent, has conciliated different views on the regulation and function of APCs in AT.

It's also debatable about the phenotypes and function of Th17 cells in obesity development. Most studies suggest that HFD feeding promotes the percentages of Th17 cells in AT and periphery, which contributes to the acceleration of obesity and some autoimmune diseases in which obesity is recognized as a risk factor (10, 45, 46, 52). However, HFD-derived gut microbiota decreases Th17 cells in the lamina propria (47). Heterozygous RORγt^+/−−^ mice promote diet-induced obesity and insulin resistance compared with WT mice (47). Delivery of Th17 cells to the intestines of obese mice results in expansion of commensal microbes that maintain metabolic homeostasis (104). One explanation for the discrepancies may be due to the differences in the functions of local APCs. HFD-induced gut microbiota impairs the gene expression profile and function of lamina propria APCs required for Th17 Cell Differentiation (47), whereas the APCs in AT of obese mice show higher levels of cytokines secretion or surface markers expression that facilitate Th17 cell differentiation (12, 52). Still, there is a possibility that Th17 cells in distinct organs may function differently.

The recent groundbreaking research on the roles of AT-resident CD4^+^ T cells in the regulation of insulin sensitivity and energy homeostasis has shed new light on our understanding of the communication between immune cells and adipocytes, paving a road to the development of novel therapeutic strategies for the treatment of obesity and its associated diseases. However, many important questions remain to be addressed. Identifying the molecular nature of antigens associated with AT inflammation during obesity is of great importance, which can help to restrain proinflammatory CD4^+^ T cell immune responses. A future challenge will also involve ascertaining the possibilities and molecular mechanisms of the functional interplay between other immune cells and CD4^+^ T cells, especially in SAT and BAT. Unlike their circulating counterparts, Treg cells express an AT specific marker PPAR-γ (31) and show a high degree of adaptation to the surrounding milieu. Thus, it is assumable that the metabolic microenvironment in AT may also endow other CD4^+^ T cells with specific characters. An answer to this question would provide new insights into developing organ-specific therapies for obesity and its related metabolic disorders. Nevertheless, translating the preclinical findings into clinical applications remains a great challenge, waiting for a further understanding of the mechanisms regulating the interaction between AT-resident immune cells and adipocytes under both physiological and pathological conditions.

## Author contributions

HZ organized and wrote the draft. FL revised the whole manuscript and figures and offered constructive comments.

### Conflict of interest statement

The authors declare that the research was conducted in the absence of any commercial or financial relationships that could be construed as a potential conflict of interest.

## References

[1] Ogden CL Carroll MD Kit BK Flegal KM Prevalence of childhood and adult obesity in the United States, 2011-2012. JAMA (2014) 311:806–14. 10.1001/jama.2014.732 24570244 PMC4770258

[2] Feuerer M Herrero L Cipolletta D Naaz A Wong J Nayer A Lean, but not obese, fat is enriched for a unique population of regulatory T cells that affect metabolic parameters. Nat Med. (2009) 15:930–9. 10.1038/nm.2002 19633656 PMC3115752

[3] Rosen ED Spiegelman BM. What we talk about when we talk about fat. Cell (2014) 156:20–44. 10.1016/j.cell.2013.12.012 24439368 PMC3934003

[4] Kalin S Becker M Ott VB Serr I Hosp F Mollah MMH. A Stat6/Pten axis links regulatory T cells with adipose tissue function. Cell Metab. (2017) 26:475–92 e7. 10.1016/j.cmet.2017.08.008 28877454 PMC5627977

[5] Han SJ Glatman Zaretsky A Andrade–Oliveira V Collins N Dzutsev A Shaik J. White adipose tissue is a reservoir for memory T cells and promotes protective memory responses to infection. Immunity (2017) 47:1154–68 e6. 10.1016/j.immuni.2017.11.009 29221731 PMC5773068

[6] Mauro C Smith J Cucchi D Coe D Fu H Bonacina F. Obesity-Induced metabolic stress leads to biased effector memory CD4(+) T cell differentiation via PI3K p110delta-Akt-mediated signals. Cell Metab. (2017) 25:593–609. 10.1016/j.cmet.2017.01.008 28190771 PMC5355363

[7] Kintscher U Hartge M Hess K Foryst-Ludwig A Clemenz M Wabitsch M. T-lymphocyte infiltration in visceral adipose tissue: a primary event in adipose tissue inflammation and the development of obesity-mediated insulin resistance. Arterioscler Thromb Vasc Biol. (2008) 28:1304–10. 10.1161/ATVBAHA.108.165100 18420999

[8] Deiuliis J Shah Z Shah N Needleman B Mikami D Narula V. Visceral adipose inflammation in obesity is associated with critical alterations in tregulatory cell numbers. PLoS ONE (2011) 6:e16376. 10.1371/journal.pone.0016376 21298111 PMC3027666

[9] Rocha VZ Folco EJ Sukhova G Shimizu K Gotsman I Vernon AH. Interferon-gamma, a Th1 cytokine, regulates fat inflammation: a role for adaptive immunity in obesity. Circ Res. (2008) 103:467–76. 10.1161/CIRCRESAHA.108.177105 18658050 PMC2740384

[10] Endo Y Asou HK Matsugae N Hirahara K Shinoda K Tumes DJ. Obesity drives Th17 cell differentiation by inducing the lipid metabolic kinase, ACC1. Cell Rep. (2015) 12:1042–55. 10.1016/j.celrep.2015.07.014 26235623

[11] Cho KW Morris DL DelProposto JL Geletka L Zamarron B Martinez-Santibanez G An MHCII-dependent activation loop between adipose tissue macrophages and CD4+ T cells controls obesity-induced inflammation. Cell Rep. (2014) 9:605–17. 10.1016/j.celrep.2014.09.004 25310975 PMC4252867

[12] Bertola A Ciucci T Rousseau D Bourlier V Duffaut C Bonnafous S. Identification of adipose tissue dendritic cells correlated with obesity-associated insulin-resistance and inducing Th17 responses in mice and patients. Diabetes (2012) 61:2238–47. 10.2337/db11-1274 22596049 PMC3425417

[13] Deng T Liu J Deng Y Minze L Xiao X Wright V Adipocyte adaptive immunity mediates diet-induced adipose inflammation and insulin resistance by decreasing adipose Treg cells. Nat Commun. (2017) 8:15725. 10.1038/ncomms15725

[14] Zhu J Yamane H Paul WE. Differentiation of effector CD4 T cell populations (^*^). Annu Rev Immunol. (2010) 28:445–89. 10.1146/annurev-immunol-030409-101212 20192806 PMC3502616

[15] Zhu J Paul WE. CD4 T cells: fates, functions, and faults. Blood (2008) 112:1557–69. 10.1182/blood-2008-05-078154 18725574 PMC2518872

[16] Holgate ST. Innate and adaptive immune responses in asthma. Nat Med. (2012) 18:673–83. 10.1038/nm.2731 22561831

[17] Zhou H Wang Y Lian Q Yang B Ma Y Wu X. Differential IL-10 production by DCs determines the distinct adjuvant effects of LPS and PTX in EAE induction. Eur J Immunol. (2014) 44:1352–62. 10.1002/eji.201343744 24496948

[18] Bazzazi H Aghaei M Memarian A Asgarian-Omran H Behnampour N Yazdani Y. Th1-Th17 ratio as a new insight in Rheumatoid arthritis disease. Iran J Allergy Asthma Immunol. (2018) 17:68–77. 29512371

[19] Burzyn D Benoist C Mathis D. Regulatory T cells in nonlymphoid tissues. Nat Immunol. (2013) 14:1007–13. 10.1038/ni.2683 24048122 PMC4708287

[20] Szabo SJ Sullivan BM Stemmann C Satoskar AR Sleckman BP Glimcher LH. Distinct effects of T-bet in TH1 lineage commitment and IFN-gamma production in CD4 and CD8 T cells. Science (2002) 295:338–42. 10.1126/science.1065543 11786644

[21] Zhu J Min B Hu-Li J Watson CJ Grinberg A Wang Q. Conditional deletion of Gata3 shows its essential function in T(H)1-T(H)2 responses. Nat Immunol. (2004) 5:1157–65. 10.1038/ni1128 15475959

[22] Harrington LE Hatton RD Mangan PR Turner H Murphy TL Murphy KM. Interleukin 17-producing CD4+ effector T cells develop via a lineage distinct from the T helper type 1 and 2 lineages. Nat Immunol. (2005) 6:1123–32. 10.1038/ni1254 16200070

[23] Zheng Y Rudensky AY. Foxp3 in control of the regulatory T cell lineage. Nat Immunol. (2007) 8:457–62. 10.1038/ni1455 17440451

[24] Ilan Y Maron R Tukpah AM Maioli TU Murugaiyan G Yang K. Induction of regulatory T cells decreases adipose inflammation and alleviates insulin resistance in ob/ob mice. Proc Natl Acad Sci USA. (2010) 107:9765–70. 10.1073/pnas.0908771107 20445103 PMC2906892

[25] Lumeng CN Saltiel AR. Inflammatory links between obesity and metabolic disease. J Clin Invest. (2011) 121:2111–7. 10.1172/JCI57132 21633179 PMC3104776

[26] Boyman O Kovar M Rubinstein MP Surh CD Sprent J. Selective stimulation of T cell subsets with antibody-cytokine immune complexes. Science (2006) 311:1924–7. 10.1126/science.1122927 16484453

[27] Lynch L Michelet X Zhang S Brennan PJ Moseman A Lester C. Regulatory iNKT cells lack expression of the transcription factor PLZF and control the homeostasis of T(reg) cells and macrophages in adipose tissue. Nat Immunol. (2015) 16:85–95. 10.1038/ni.3047 25436972 PMC4343194

[28] Kolodin D van Panhuys N Li C Magnuson AM Cipolletta D Miller CM. Antigen- and cytokine-driven accumulation of regulatory T cells in visceral adipose tissue of lean mice. Cell Metab. (2015) 21:543–57. 10.1016/j.cmet.2015.03.005 25863247 PMC4747251

[29] Pichery M Mirey E Mercier P Lefrancais E Dujardin A Ortega N. Endogenous IL-33 is highly expressed in mouse epithelial barrier tissues, lymphoid organs, brain, embryos, and inflamed tissues: *in situ* analysis using a novel Il-33-LacZ gene trap reporter strain. J Immunol. (2012) 188:3488–95. 10.4049/jimmunol.1101977 22371395

[30] Molofsky AB Van Gool F Liang HE Van Dyken SJ Nussbaum JC Lee J. Interleukin-33 and interferon-gamma counter-regulate group 2 innate lymphoid cell activation during immune perturbation. Immunity (2015) 43:161–74. 10.1016/j.immuni.2015.05.019 26092469 PMC4512852

[31] Cipolletta D Feuerer M Li A Kamei N Lee J Shoelson SE. PPAR-gamma is a major driver of the accumulation and phenotype of adipose tissue Treg cells. Nature (2012) 486:549–53. 10.1038/nature11132 22722857 PMC3387339

[32] Molofsky AB Nussbaum JC Liang HE Van Dyken SJ Cheng LE Mohapatra A. Innate lymphoid type 2 cells sustain visceral adipose tissue eosinophils and alternatively activated macrophages. J Exp Med. (2013) 210:535–49. 10.1084/jem.20121964 23420878 PMC3600903

[33] Miller AM Asquith DL Hueber AJ Anderson LA Holmes WM McKenzie AN. Interleukin-33 induces protective effects in adipose tissue inflammation during obesity in mice. Circ Res. (2010) 107:650–8. 10.1161/CIRCRESAHA.110.218867 20634488 PMC4254700

[34] Winer S Chan Y Paltser G Truong D Tsui H Bahrami J. Normalization of obesity-associated insulin resistance through immunotherapy. Nat Med. (2009) 15:921–9. 10.1038/nm.2001 19633657 PMC3063199

[35] Schipper HS Prakken B Kalkhoven E Boes M. Adipose tissue-resident immune cells: key players in immunometabolism. Trends Endocrinol Metab. (2012) 23:407–15. 10.1016/j.tem.2012.05.011 22795937

[36] Lumeng CN Bodzin JL Saltiel AR. Obesity induces a phenotypic switch in adipose tissue macrophage polarization. J Clin Invest. (2007) 117:175–84. 10.1172/JCI29881 17200717 PMC1716210

[37] Nishimura S Manabe I Nagasaki M Eto K Yamashita H Ohsugi M. CD8+ effector T cells contribute to macrophage recruitment and adipose tissue inflammation in obesity. Nat Med. (2009) 15:914–20. 10.1038/nm.1964 19633658

[38] Strissel KJ DeFuria J Shaul ME Bennett G Greenberg AS Obin MS. T-cell recruitment and Th1 polarization in adipose tissue during diet-induced obesity in C57BL/6 mice. Obesity (Silver Spring) (2010) 18:1918–25. 10.1038/oby.2010.1 20111012 PMC2894258

[39] Stolarczyk E Vong CT Perucha E Jackson I Cawthorne MA Wargent ET. Improved insulin sensitivity despite increased visceral adiposity in mice deficient for the immune cell transcription factor T-bet. Cell Metab. (2013) 17:520–33. 10.1016/j.cmet.2013.02.019 23562076 PMC3685808

[40] Martinez FO Gordon S. The M1 and M2 paradigm of macrophage activation: time for reassessment. F1000Prime Rep. (2014) 6:13. 10.12703/P6-13 24669294 PMC3944738

[41] Lighvani AA Frucht DM Jankovic D Yamane H Aliberti J Hissong BD. T-bet is rapidly induced by interferon-gamma in lymphoid and myeloid cells. Proc Natl Acad Sci USA. (2001) 98:15137–42. 10.1073/pnas.261570598 11752460 PMC64996

[42] Deng T Lyon CJ Minze LJ Lin J Zou J Liu JZ. Class II major histocompatibility complex plays an essential role in obesity-induced adipose inflammation. Cell Metab. (2013) 17:411–22. 10.1016/j.cmet.2013.02.009 23473035 PMC3619392

[43] Fabbrini E Cella M McCartney SA Fuchs A Abumrad NA Pietka TA. Association between specific adipose tissue CD4+ T-cell populations and insulin resistance in obese individuals. Gastroenterology (2013) 145:366–74 e1-3. 10.1053/j.gastro.2013.04.010 23597726 PMC3756481

[44] O'Rourke RW Lumeng CN. Obesity heats up adipose tissue lymphocytes. Gastroenterology (2013) 145:282–5. 10.1053/j.gastro.2013.06.026 23806542 PMC4181357

[45] Chuang HC Sheu WH Lin YT Tsai CY Yang CY Cheng YJ. HGK/MAP4K4 deficiency induces TRAF2 stabilization and Th17 differentiation leading to insulin resistance. Nat Commun. (2014) 5:4602. 10.1038/ncomms5602 25098764 PMC4143962

[46] Jhun J-Y Yoon B-Y Park M-K Oh H-J Byun J-K Lee S-Y. Obesity aggravates the joint inflammation in a collagen-induced arthritis model through deviation to Th17 differentiation. Exp Mol Med. (2012) 44:424. 10.3858/emm.2012.44.7.047 22513335 PMC3406287

[47] Garidou L Pomie C Klopp P Waget A Charpentier J Aloulou M. The gut microbiota regulates intestinal CD4 T cells expressing RORgammat and controls metabolic disease. Cell Metab. (2015) 22:100–12. 10.1016/j.cmet.2015.06.001 26154056

[48] Shin JH Shin DW Noh M Interleukin-17A inhibits adipocyte differentiation in human mesenchymal stem cells and regulates proinflammatory responses in adipocytes. Biochem Pharmacol. (2009) 77:1835–44. 10.1016/j.bcp.2009.03.008 19428338

[49] Zuniga LA Shen WJ Joyce-Shaikh B Pyatnova EA Richards AG Thom C. IL-17 regulates adipogenesis, glucose homeostasis, and obesity. J Immunol. (2010) 185:6947–59. 10.4049/jimmunol.1001269 21037091 PMC3001125

[50] Kohlgruber AC Gal-Oz ST LaMarche NM Shimazaki M Duquette D Nguyen HN. γ*δ* T cells producing interleukin-17A regulate adipose regulatory T cell homeostasis and thermogenesis. Nat Immunol. (2018) 19:464–74. 10.1038/s41590-018-0094-2 29670241 PMC8299914

[51] Morris DL Cho KW Delproposto JL Oatmen KE Geletka LM Martinez-Santibanez G. Adipose tissue macrophages function as antigen-presenting cells and regulate adipose tissue CD4+ T cells in mice. Diabetes (2013) 62:2762–72. 10.2337/db12-1404 23493569 PMC3717880

[52] Chen Y Tian J Tian X Tang X Rui K Tong J. Adipose tissue dendritic cells enhances inflammation by prompting the generation of Th17 cells. PLoS ONE (2014) 9:e92450. 10.1371/journal.pone.0092450 24642966 PMC3958510

[53] Odegaard JI Chawla A. Alternative macrophage activation and metabolism. Annu Rev Pathol. (2011) 6:275–97. 10.1146/annurev-pathol-011110-130138 21034223 PMC3381938

[54] Moraes-Vieira PM Yore MM Dwyer PM Syed I Aryal P Kahn BB. RBP4 activates antigen-presenting cells, leading to adipose tissue inflammation and systemic insulin resistance. Cell Metab. (2014) 19:512–26. 10.1016/j.cmet.2014.01.018 24606904 PMC4078000

[55] Morris DL Oatmen KE Mergian TA Cho KW DelProposto JL Singer K. CD40 promotes MHC class II expression on adipose tissue macrophages and regulates adipose tissue CD4+ T cells with obesity. J Leukoc Biol. (2016) 99:1107–19. 10.1189/jlb.3A0115-009R 26658005 PMC4952010

[56] Hill DA Lim HW Kim YH Ho WY Foong YH Nelson VL. Distinct macrophage populations direct inflammatory versus physiological changes in adipose tissue. Proc Natl Acad Sci USA. (2018) 115:E5096–105. 10.1073/pnas.1802611115 29760084 PMC5984532

[57] Zhang SY Lv Y Zhang H Gao S Wang T Feng J. Adrenomedullin 2 improves early obesity-induced adipose insulin resistance by inhibiting the class II MHC in adipocytes. Diabetes (2016) 65:2342–55. 10.2337/db15-1626 27207558

[58] Oh DY Morinaga H Talukdar S Bae EJ Olefsky JM. Increased macrophage migration into adipose tissue in obese mice. Diabetes (2012) 61:346–54. 10.2337/db11-0860 22190646 PMC3266418

[59] Kim D Kim J Yoon JH Ghim J Yea K Song P. CXCL12 secreted from adipose tissue recruits macrophages and induces insulin resistance in mice. Diabetologia (2014) 57:1456–65. 10.1007/s00125-014-3237-5 24744121

[60] Poggi M Jager J Paulmyer-Lacroix O Peiretti F Gremeaux T Verdier M. The inflammatory receptor CD40 is expressed on human adipocytes: contribution to crosstalk between lymphocytes and adipocytes. Diabetologia (2009) 52:1152–63. 10.1007/s00125-009-1267-1 19183933

[61] Duffaut C Zakaroff-Girard A Bourlier V Decaunes P Maumus M Chiotasso P. Interplay between human adipocytes and T lymphocytes in obesity: CCL20 as an adipochemokine and T lymphocytes as lipogenic modulators. Arterioscler Thromb Vasc Biol. (2009) 29:1608–14. 10.1161/ATVBAHA.109.192583 19644053

[62] De Rosa V Procaccini C Cali G Pirozzi G Fontana S Zappacosta S A key role of leptin in the control of regulatory T cell proliferation. Immunity (2007) 26:241–55. 10.1016/j.immuni.2007.01.011 17307705

[63] Matarese G Di Giacomo A Sanna V Lord GM Howard JK Di Tuoro A. Requirement for leptin in the induction and progression of autoimmune encephalomyelitis. J Immunol. (2001) 166:5909–16. 10.4049/jimmunol.166.10.5909 11342605

[64] Zeyda M Wernly B Demyanets S Kaun C Hammerle M Hantusch B. Severe obesity increases adipose tissue expression of interleukin-33 and its receptor ST2, both predominantly detectable in endothelial cells of human adipose tissue. Int J Obes. (2013) 37:658–65. 10.1038/ijo.2012.118 22828942

[65] Zheng H Zhang X Castillo EF Luo Y Liu M Yang XO. Leptin enhances TH2 and ILC2 responses in allergic airway disease. J Biol Chem. (2016) 291:22043–52. 10.1074/jbc.M116.743187 27566543 PMC5063987

[66] Reis BS Lee K Fanok MH Mascaraque C Amoury M Cohn LB. Leptin receptor signaling in T cells is required for Th17 differentiation. J Immunol. (2015) 194:5253–60. 10.4049/jimmunol.1402996 25917102 PMC4433844

[67] Yu Y Liu Y Shi FD Zou H Matarese G La Cava A. Cutting edge: leptin-induced RORgammat expression in CD4+ T cells promotes Th17 responses in systemic lupus erythematosus. J Immunol. (2013) 190:3054–8. 10.4049/jimmunol.1203275 23447682 PMC3608794

[68] Lord GM Matarese G Howard JK Baker RJ Bloom SR Lechler RI. Leptin modulates the T-cell immune response and reverses starvation-induced immunosuppression. Nature (1998) 394:897–901. 10.1038/29795 9732873

[69] Cheng X Folco EJ Shimizu K Libby P Adiponectin induces proinflammatory programs in human macrophages and CD4+ T cells. J Biol Chem. (2012) 287:36896–904. 10.1074/jbc.M112.409516 22948153 PMC3481292

[70] Jung MY Kim HS Hong HJ Youn BS Kim TS. Adiponectin induces dendritic cell activation via PLCgamma/JNK/NF-kappaB pathways, leading to Th1 and Th17 polarization. J Immunol. (2012) 188:2592–601. 10.4049/jimmunol.1102588 22345647

[71] Walcher D Hess K Berger R Aleksic M Heinz P Bach H. Resistin: A newly identified chemokine for human CD4-positive lymphocytes. Cardiovasc Res. (2010) 85:167–74. 10.1093/cvr/cvp278 19684036

[72] Haus JM Kashyap SR Kasumov T Zhang R Kelly KR Defronzo RA. Plasma ceramides are elevated in obese subjects with type 2 diabetes and correlate with the severity of insulin resistance. Diabetes (2009) 58:337–43. 10.2337/db08-1228 19008343 PMC2628606

[73] Kue CS Jung MY Cho D Kim TS. C6-ceramide enhances Interleukin-12-mediated T helper type 1 cell responses through a cyclooxygenase-2-dependent pathway. Immunobiology (2012) 217:601–9. 10.1016/j.imbio.2011.10.021 22112438

[74] Park J Li Q Chang YT Kim TS. Inhibitory activity of a ceramide library on interleukin-4 production from activated T cells. Bioorg Med Chem. (2005) 13:2589–95. 10.1016/j.bmc.2005.01.027 15755660

[75] Hou TY McMurray DN Chapkin RS. Omega-3 fatty acids, lipid rafts, and T cell signaling. Eur J Pharmacol. (2016) 785:2–9. 10.1016/j.ejphar.2015.03.091 26001374 PMC4654711

[76] Monk JM Hou TY Turk HF Weeks B Wu C McMurray DN. Dietary n-3 polyunsaturated fatty acids (PUFA) decrease obesity-associated Th17 cell-mediated inflammation during colitis. PLoS ONE (2012) 7:e49739. 10.1371/journal.pone.0049739 23166761 PMC3500317

[77] Banchereau J Steinman RM. Dendritic cells and the control of immunity. Nature (1998) 392:245–52. 10.1038/32588 9521319

[78] Cho KW Zamarron BF Muir LA Singer K Porsche CE DelProposto JB. Adipose tissue dendritic cells are independent contributors to obesity-induced inflammation and insulin resistance. J Immunol. (2016) 197:3650–61. 10.4049/jimmunol.1600820 27683748 PMC5555636

[79] Patsouris D Li P-P Thapar D Chapman J Olefsky JM Neels JG. Ablation of CD11c-positive cells normalizes insulin sensitivity in obese insulin resistant animals. Cell Metab. (2008) 8:301–9. 10.1016/j.cmet.2008.08.015 18840360 PMC2630775

[80] Poltorak MP Schraml BU. Fate mapping of dendritic cells. Front Immunol. (2015) 6:199. 10.3389/fimmu.2015.00199 25999945 PMC4418393

[81] Coombes JL Siddiqui KR Arancibia-Carcamo CV Hall J Sun CM Belkaid Y. A functionally specialized population of mucosal CD103+ DCs induces Foxp3+ regulatory T cells via a TGF-beta and retinoic acid-dependent mechanism. J Exp Med. (2007) 204:1757–64. 10.1084/jem.20070590 17620361 PMC2118683

[82] Medrikova D Sijmonsma TP Sowodniok K Richards DM Delacher M Sticht C. Brown adipose tissue harbors a distinct sub-population of regulatory T cells. PLoS ONE (2015) 10:e0118534. 10.1371/journal.pone.0118534 25714366 PMC4340926

[83] Powell JD Pollizzi KN Heikamp EB Horton MR. Regulation of immune responses by mTOR. Annu Rev Immunol. (2012) 30:39–68. 10.1146/annurev-immunol-020711-075024 22136167 PMC3616892

[84] Walker JA Barlow JL McKenzie AN. Innate lymphoid cells–how did we miss them?. Nat Rev Immunol. (2013) 13:75–87. 10.1038/nri3349 23292121

[85] Liu X Huh JY Gong H Chamberland JP Brinkoetter MT Hamnvik OP. Lack of mature lymphocytes results in obese but metabolically healthy mice when fed a high-fat diet. Int J Obes. (2015) 39:1548–57. 10.1038/ijo.2015.93 25994806 PMC5321118

[86] Moysidou M Karaliota S Kodela E Salagianni M Koutmani Y Katsouda A. CD8+ T cells in beige adipogenesis and energy homeostasis. JCI Insight (2018) 3:95456. 10.1172/jci.insight.95456 29515042 PMC5922290

[87] Perez-Martinez L Perez-Matute P Aguilera-Lizarraga J Rubio-Mediavilla S Narro J Recio E. Maraviroc, a CCR5 antagonist, ameliorates the development of hepatic steatosis in a mouse model of non-alcoholic fatty liver disease (NAFLD). J Antimicrob Chemother. (2014) 69:1903–10. 10.1093/jac/dku071 24651825

[88] Wu H Ghosh S Perrard XD Feng L Garcia GE Perrard JL. T-cell accumulation and regulated on activation, normal T cell expressed and secreted upregulation in adipose tissue in obesity. Circulation (2007) 115:1029–38. 10.1161/CIRCULATIONAHA.106.638379 17296858

[89] Bigorgne AE Bouchet-Delbos L Naveau S Dagher I Prevot S Durand-Gasselin I. Obesity-induced lymphocyte hyperresponsiveness to chemokines: a new mechanism of Fatty liver inflammation in obese mice. Gastroenterology (2008) 134:1459–69. 10.1053/j.gastro.2008.02.055 18471520

[90] Matter CM Handschin C. RANTES (regulated on activation, normal T cell expressed and secreted), inflammation, obesity, and the metabolic syndrome. Circulation (2007) 115:946–8. 10.1161/CIRCULATIONAHA.106.685230 17325252

[91] Bachelerie F Ben-Baruch A Burkhardt AM Combadiere C Farber JM Graham GJ. International union of basic and clinical pharmacology. [corrected]. LXXXIX. Update on the extended family of chemokine receptors and introducing a new nomenclature for atypical chemokine receptors. Pharmacol Rev. (2014) 66:1–79. 10.1124/pr.113.007724 24218476 PMC3880466

[92] Conroy MJ Galvin KC Kavanagh ME Mongan AM Doyle SL Gilmartin N. CCR1 antagonism attenuates T cell trafficking to omentum and liver in obesity-associated cancer. Immunol Cell Biol. (2016) 94:531–7. 10.1038/icb.2016.26 27046081

[93] Biddinger SB Kahn CR. From mice to men: insights into the insulin resistance syndromes. Annu Rev Physiol. (2006) 68:123–58. 10.1146/annurev.physiol.68.040104.124723 16460269

[94] Luczynski W Wawrusiewicz-Kurylonek N Ilendo E Bossowski A Glowinska-Olszewska B Kretowski A. Generation of functional T-regulatory cells in children with metabolic syndrome. Arch Immunol Ther Exp. (2012) 60:487–95. 10.1007/s00005-012-0198-6 23052042

[95] Belghith M Bluestone JA Barriot S Megret J Bach JF Chatenoud L. TGF-beta-dependent mechanisms mediate restoration of self-tolerance induced by antibodies to CD3 in overt autoimmune diabetes. Nat Med. (2003) 9:1202–8. 10.1038/nm924 12937416

[96] Chatenoud L Bluestone JA. CD3-specific antibodies: a portal to the treatment of autoimmunity. Nat Rev Immunol. (2007) 7:622–32. 10.1038/nri2134 17641665

[97] Daniel C Wennhold K Kim HJ von Boehmer H. Enhancement of antigen-specific Treg vaccination *in vivo*. Proc Natl Acad Sci USA. (2010) 107:16246–51. 10.1073/pnas.1007422107 20805478 PMC2941325

[98] Mozaffarian D Hao T Rimm EB Willett WC Hu FB. Changes in diet and lifestyle and long-term weight gain in women and men. N Engl J Med. (2011) 364:2392–404. 10.1056/NEJMoa1014296 21696306 PMC3151731

[99] Kim KA Gu W Lee IA Joh EH Kim DH. High fat diet-induced gut microbiota exacerbates inflammation and obesity in mice via the TLR4 signaling pathway. PLoS ONE (2012) 7:e47713. 10.1371/journal.pone.0047713 23091640 PMC3473013

[100] Chi Y Lin Y Zhu H Huang Q Ye G Dong S. PCBs-high-fat diet interactions as mediators of gut microbiota dysbiosis and abdominal fat accumulation in female mice. Environ Pollut. (2018) 239:332–41. 10.1016/j.envpol.2018.04.001 29674211

[101] Ley RE Turnbaugh PJ Klein S Gordon JI. Microbial ecology: human gut microbes associated with obesity. Nature (2006) 444:1022–3. 10.1038/4441022a 17183309

[102] Poutahidis T Kleinewietfeld M Smillie C Levkovich T Perrotta A Bhela S. Microbial reprogramming inhibits Western diet-associated obesity. PLoS ONE (2013) 8:e68596. 10.1371/journal.pone.0068596 23874682 PMC3707834

[103] Roselli M Devirgiliis C Zinno P Guantario B Finamore A Rami R. Impact of supplementation with a food-derived microbial community on obesity-associated inflammation and gut microbiota composition. Genes Nutr. (2017) 12:25. 10.1186/s12263-017-0583-1 29043005 PMC5628415

[104] Hong CP Park A Yang BG Yun CH Kwak MJ Lee GW. Gut-specific delivery of T-Helper 17 cells reduces obesity and insulin resistance in mice. Gastroenterology (2017) 152:1998–2010. 10.1053/j.gastro.2017.02.016 28246016

